# The risk ethics of autonomous vehicles: an empirical approach

**DOI:** 10.1038/s41598-024-51313-2

**Published:** 2024-01-10

**Authors:** Sebastian Krügel, Matthias Uhl

**Affiliations:** 1 Faculty of Computer Science, Technische Hochschule Ingolstadt, Esplanade 10, 85049 Ingolstadt, Germany; 2 TUM School of Social Sciences and Technology, Technical University of Munich, Richard-Wagner-Str. 1, 80333 Munich, Germany

**Keywords:** Psychology, Engineering

## Abstract

How would people distribute risks of autonomous vehicles (AVs) in everyday road traffic? The rich literature on the ethics of autonomous vehicles (AVs) revolves around moral judgments in unavoidable collision scenarios. We argue for extending the debate to driving behaviors in everyday road traffic where ubiquitous ethical questions arise due to the permanent redistribution of risk among road users. This distribution of risks raises ethically relevant questions that cannot be evaded by simple heuristics such as “hitting the brakes.” Using an interactive, graphical representation of different traffic situations, we measured participants’ preferences on driving maneuvers of AVs in a representative survey in Germany. Our participants’ preferences deviated significantly from mere collision avoidance. Interestingly, our participants were willing to take risks themselves for the benefit of other road users, suggesting that the social dilemma of AVs may be mitigated in risky environments. Our research might build a bridge between engineers and philosophers to discuss the ethics of AVs more constructively.

## Introduction

Deterministic dilemmas resembling the trolley problem^1^ still predominate ethical discussions on AVs. This neglects the fact that road traffic is not deterministic, but associated with varying levels of risk dependent on numerous factors such as weather conditions, road users’ behavior, technical malfunctions and so on^2–6^. Connected AVs may be a chance for managing traffic risk more deliberately than impulse-driven manual traffic allows. By focusing on unavoidable collisions, however, the recent ethical perspective sets collision probability to one and solely discusses collision severity. Conversely, the engineering perspective is essentially guided by collision avoidance^7–10^, and therefore it questions the relevance of the ethics of unavoidable collisions^4,11^. The focus of engineering is on minimizing collision probability. Both perspectives neglect the fact that any maneuver in everyday road traffic constitutes a redistribution of risks that is a function of both collision probability and collision severity. One obvious determinant of collision severity is the number of passengers in any given vehicle. In a future with connected driving, this information could be exchanged between vehicles.

Until recently, moral philosophy has not systematically addressed the ethical issues of risk. The complexities arising from a non-deterministic world have traditionally been dealt with in the domain of decision theory, while ethics has dealt with well-defined situations. Risk ethics recognizes that with the imposition of risk, difficult problems arise that cannot be evaded simply by the probabilistic mixture of potential outcomes^12^. This may be illustrated by a simple example. A utilitarian clearly prefers the certain death of one person to the certain death of two. However, it is not so clear whether a utilitarian also prefers the risk of one person dying with probability one half to the risk of two persons dying with probability one third. This is because utilitarianism is compatible with several ways of evaluating uncertain events and the expected-utility criterion being only one of them^12^.

That these issues are not merely part of an academic mock debate becomes evident when we look at industrial research on AVs. In a series of patents^13–15^, *Google Inc.* and later *Waymo LLC*, for instance, set out how AVs can evaluate different actions based on a risk-cost framework. This framework adopts the expected-utility criterion, which compares available actions based on the respective sum of risk penalties, each determined as the product of risk magnitude and probability. Although it remains open how (and by whom) the risk magnitude of a bad event is quantified, the risks for other road users (e.g., pedestrians) are explicitly considered in the AV’s risk management. In yet another patent^16^, *Google Inc.* describes the possibility of adjusting lateral lane positioning of an AV depending on the respective traffic situation. On the one hand, it is envisaged that an AV could enhance its own safety by increasing the lateral distance to a larger object (e.g., truck) on one side, even if this reduces the lateral distance to a smaller object (e.g., small car) on the other side of the road. On the other hand, the AV might also consider the vulnerability of other road users and generally maintain more lateral distance to them. Clearly, AV manufacturers might implement, either consciously or unconsciously, various risk ethical approaches to AV driving behavior, each of which could be challenged on a normative basis.

With the possible deployment of AVs on the road, questions of risk ethics are therefore unavoidable and any society considering their use must face a debate about the appropriate risk approach. To enrich the ethical debate on the ethics of unavoidable crashes, laypeople’s choices in trolley problems with AVs have been elicited^17–21^. These results can be transferred to everyday traffic situations if and only if one interprets them as statistical trolleys^22^. If a certain traffic situation occurs a million times, collisions will be unavoidable in the aggregate. This view, however, neglects the potential trade-off between the probability and severity of a collision.

In the present paper, we therefore investigate individual choices under risk explicitly by letting people make moral decisions in mundane and non-deterministic road traffic. By looking at safety distances, we study the rationale by which people manage the trade-offs between collision probability and collision severity without assuming one of the components away. Driving algorithms factually distribute risks in road traffic among different road users when determining the safety distance between them^6,21–23^. This is also acknowledged by the European Commission’s report on the Ethics of Connected and Automated Vehicles^24^. Specifically, recommendation 5 requests AVs to adapt their behavior around vulnerable road users, while recommendation 6 emphasizes the ethical implications emerging from AVs’ continuous statistical distribution of risk in the pursuit of safety and equality. Reflecting on the specific principles underlying this distribution of risk through an ethical discourse seems more sensible than leaving it to the (explicit or implicit) judgments of car manufacturers^13–16^.

## Study design

**Figure 1:**
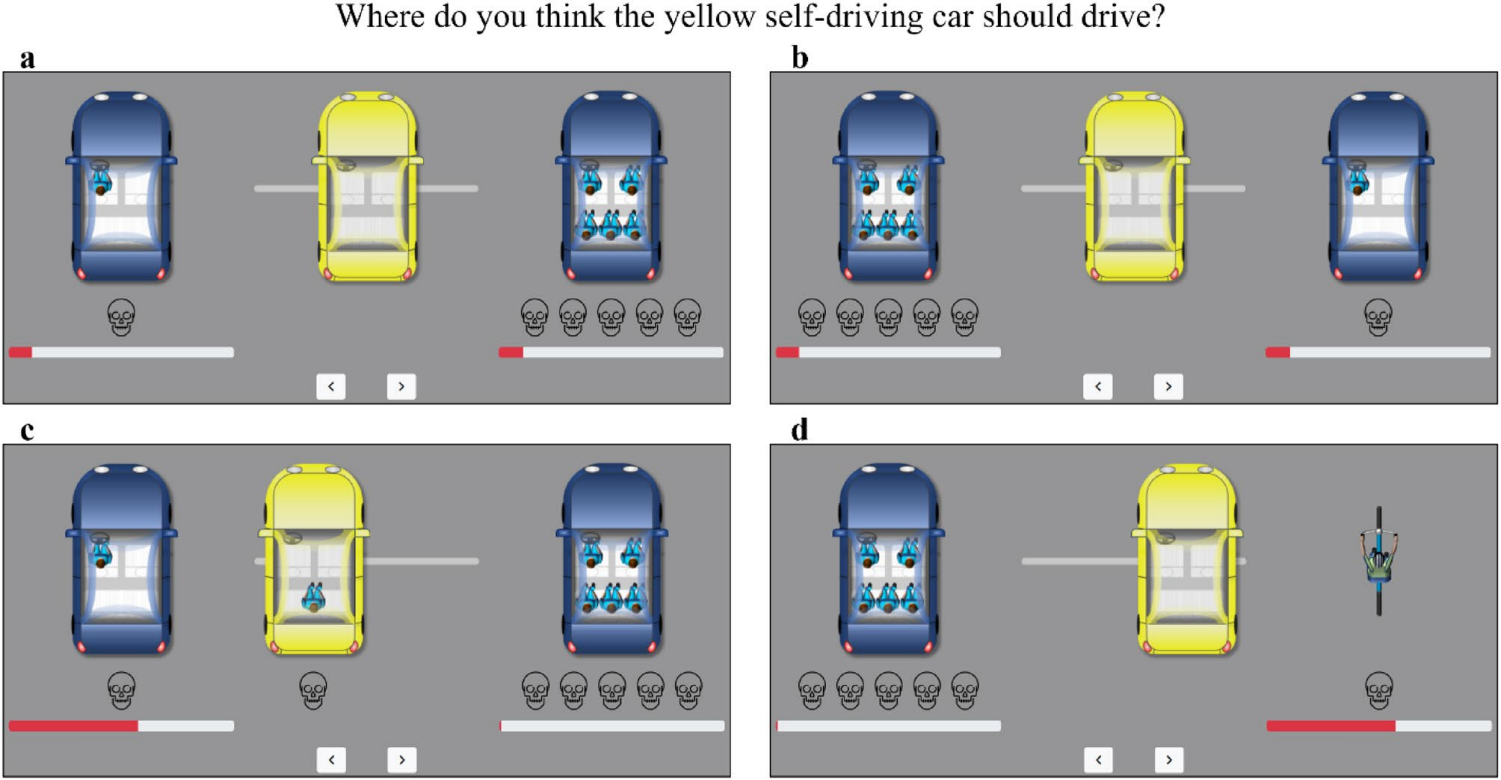
Graphical interface for eliciting preferences about risk allocation in road traffic. The figure shows four traffic situations of a self-driving car. The probability of collision with another road user was very small, but not zero. The smaller the distance, the higher the probability of a collision. If a collision occurred, all involved parties were dead. The self-driving car could not collide with both other road users simultaneously.

To elicit laypeople’s intuitions about the distribution of risk in everyday road traffic, we developed a graphical interface depicting a common traffic situation in a future with AVs operating in mixed traffic. Figure 1 exemplifies four traffic situations out of a total of 29 used in our study. In all situations, a self-driving (yellow) car was depicted between two other road users. Participants could gradually adjust its driving position between them in 99 increments by dragging the yellow car to the left or right or by using the arrow keys below it. Participants were told that the overall probability of a collision was very small, but not zero, and that the probability of a collision with either vehicle depended on the safety distance of the yellow car. The smaller this distance, the greater the probability of a collision with that vehicle, assuming that the yellow car cannot collide with both vehicles simultaneously. Furthermore, it should be presumed that, in the event of a collision, all parties involved in the collision would be dead.

We presented the task of positioning the AV to a representative sample of 1807 participants in Germany. Each participant assessed only one traffic situation, and the initial position of the yellow car was always chosen at random. We varied the traffic situations along three dimensions: first, the number of passengers in the other two cars to the left and right of the yellow AV; second, the type of vehicle on the right side of the road (a car with one passenger or one cyclist); and, finally, whether or not the participants themselves were part of the traffic situation by being passengers in the yellow AV. Except for the treatments with a cyclist, we also mirrored each traffic situation so that more passengers were sometimes shown on the left and sometimes on the right side of the road.

To highlight the possible rationale of collision avoidance in our traffic situations graphically, we visualized the collision probability with the left or right vehicle using red bars below the respective vehicle (see Fig. 1). The middle driving position of the AV between the two other vehicles minimized the overall collision probability. This probability grew exponentially with deviations from the middle driving position. The shorter the distance to one of the two other vehicles, the greater the increase in the probability of a collision with this vehicle was.

## Results

### Influence of the number of other road users

**Figure 2:**
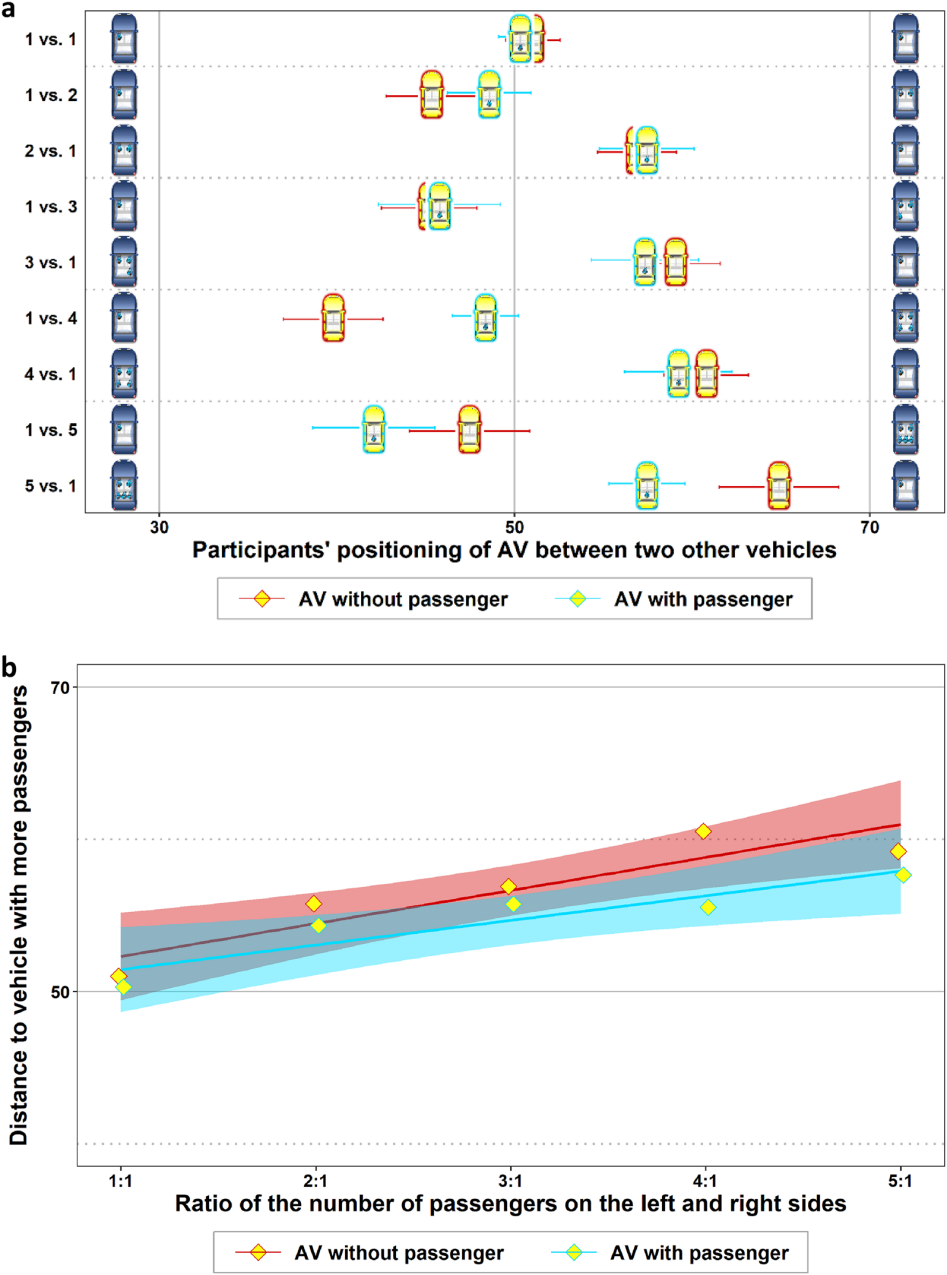
Risk allocation between road users of the same type. (**a**) Means and standard errors of participants’ positioning of the AV with different numbers of passengers in the blue cars on the left and right sides. AVs with red frames depict the results when the AVs were empty; those with blue frames show the results when the participants were passengers in the AV. (**b**) The results of a linear regression in which the AV’s distance to the car with more passengers was regressed on the increasing disproportion of passengers on the left and right sides. The disproportion of passengers was interacted with a dummy variable for the treatment (passenger in AV yes/no). Red and blue regions visualize 95% CIs.

Figure 2a shows the participants’ average positioning of the AV between the two other cars, where the number of passengers in the latter varied. Focusing first on the situations in which the participants were not part of the traffic situation [see the AVs with red frames (= treatment *AV without passenger*)], it is evident that, on average, participants considered the number of passengers in the two other cars in their AV positioning. Comparing each of the pairwise mirrored traffic situations that differed only in terms of whether more passengers appeared in the cars on the left or right side of the road, the average driving position of the AV was significantly different in all of these paired situations. On average, participants always positioned the AV closer to the car with fewer passengers (*t* > 3.4, *p* < 0.0011 for each of the paired situations). While participants placed the AV virtually in the middle driving position when there was one passenger in each car on both sides of the road, the driving position was adjusted according to the greater number of passengers in all other situations. The red line in Fig. 2b visualizes a significant positive trend between the AV’s safety distance to the car with more passengers and the disproportion of passengers on both sides (*p* < 0.001, see regression (1) in Table 1).

**Table 1:** Coefficients and, in parentheses, standard errors of regressions on which Fig. 2b is based.

	Dependent variable: *distance to more passengers*		
	*(1)*	*(2)*	*(3)*
	*AV without passenger*	*AV with passenger*	*AV without vs. with passenger*
*Constant*	50.14*** (1.82)	49.84*** (1.58)	50.14*** (1.82)
*Ratio of passengers*	2.17*** (0.63)	1.62** (0.52)	2.17*** (0.63)
*AV with passenger* (= 1)			− 0.30 (2.41)
*(AV with passenger)* × *(ratio of passengers)*			− 0.55 (0.81)
Observations	492	489	981
Log likelihood	− 2139.88	− 2110.93	− 4251.07
AIC	4285.8	4227.9	8512.1

*p < 0.05 **p < 0.01 ***p < 0.001.

Surprisingly, the results were very similar when participants had to imagine that they themselves were part of the traffic situation [see AVs with blue frames (= treatment *AV with passenger*)]. Again, the AVs’ average driving position always differed significantly between the mirrored traffic situations and was always closer to the car with fewer passengers (*t* > 2.49, *p* < 0.015 for each of the paired situations). Overall, we see again a significant positive trend between the AV’s safety distance to the car with more passengers and the disproportion of passengers in the two other cars (see the blue line in Fig. 2b and regression (2) in Table 1). In fact, the two trend lines in Fig. 2b suggest that there were no significant differences between the treatments where participants were passengers of the AV and where they were only neutral observers of the situation (see also regression (3) in Table 1). This is surprising, because previous studies with deterministic trolley problems suggested that people prefer riding with AVs that protect them as passengers at all costs^17^.

The comparisons between the two treatments *AV without* and *AV with passenger* in Fig. 2a,b are based on situations with one passenger sitting in a blue car on one side of the road and one, two, three, four or five passengers sitting in the other blue car on the opposite side of the road. The only difference between the two treatments was that in one treatment there was a passenger (i.e., the respective participant) sitting in the AV and in the other treatment the AV was empty. It might be argued that the comparison of the two treatments *AV without* and *AV with passenger* in these situations is problematic, because it ignores the passenger in the AV as a victim of the collision. For example, assume the AV with passenger drives between two cars, with two passengers in the left car and one passenger in the right car of the road (i.e., 2 vs. 1). Then, the comparison based on the actual number of collision victims is three in a collision of the AV with the left car and two in a collision of the AV with the right car of the road. Thus, the passenger inside the AV is added to the number of passengers in the cars on the left and right sides of the road under this rationale, rather than being ignored as a collision victim.

**Figure 3:**
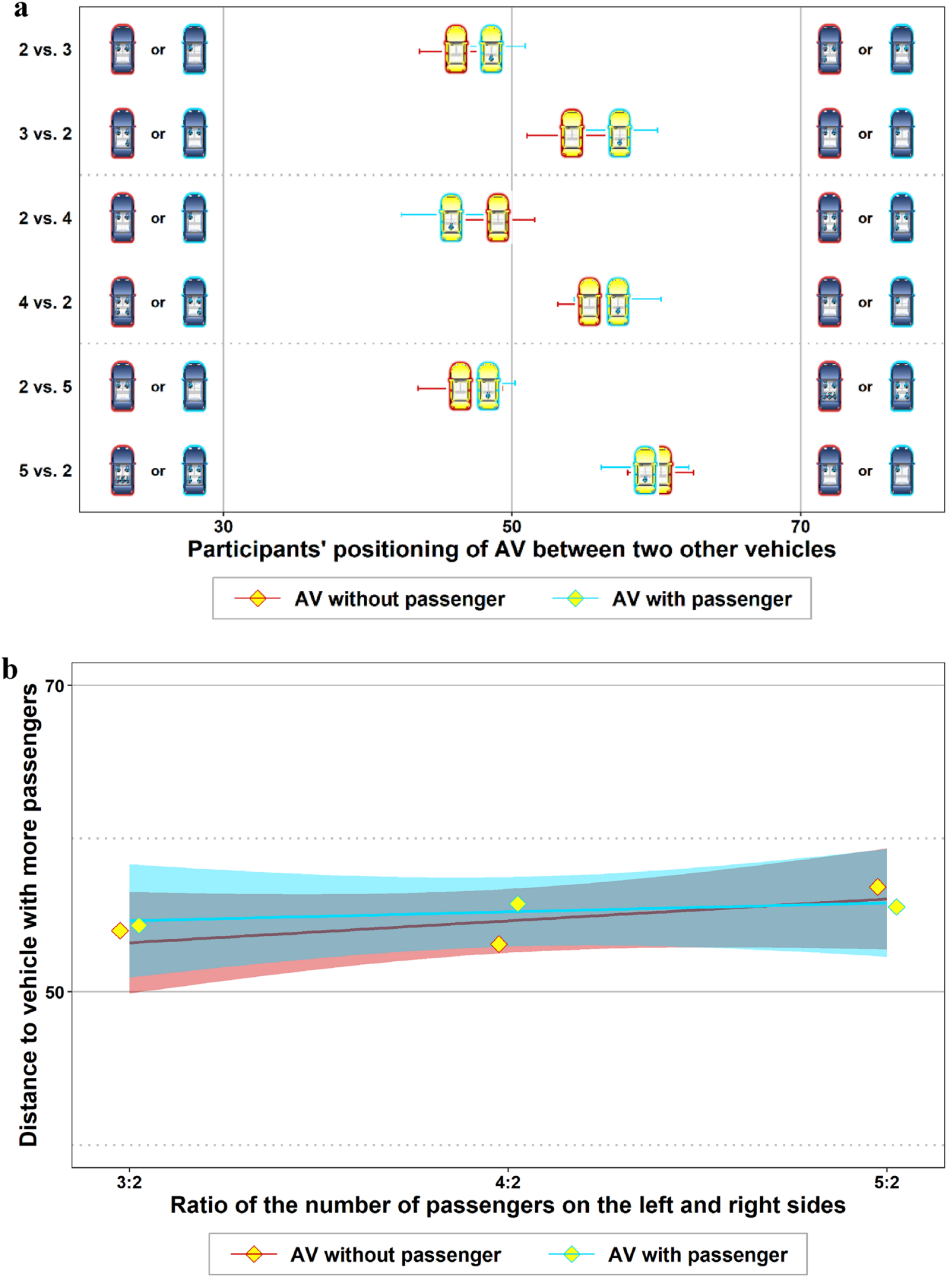
Risk allocation between road users of the same type when the passenger of the AV is taken into account as a collision victim. (**a**) Means and standard errors of participants’ positioning of the AV with different numbers of passengers in the blue cars on the left and right sides. Red frames depict the results when the AVs were empty; those with blue frames show the results where the participants were a passenger in the AV. (**b**) The results of a linear regression in which the AV’s distance from the car with more passengers was regressed on the increasing disproportion of passengers on the left and right sides. The disproportion of passengers was interacted with a dummy variable for the treatment (passenger in AV yes/no). Red and blue regions visualize 95% CIs.

For this reason, we had included additional traffic situations in the treatment *AV without passenger* in our study that enabled this comparison. That is, for the comparison with the situations 2 vs. 1, 3 vs. 1 and 4 vs. 1 in the treatment *AV with passenger*, we added the situations 3 vs. 2, 4 vs. 2 and 5 vs. 2 in the treatment *AV without passenger*. Now, the number of collision victims is the same in both treatments if the passenger in the AV is taken into account in each case. Figure 3a shows the mean driving positions of the participants in the treatments *AV without* and *AV with passenger* under this rationale. In none of the pairwise comparisons did we find a significant difference between *AV without* and *AV with passenger* (*p* > 0.42 in all pairwise *t*-tests). The two trend lines in Fig. 3b do not show a significant difference between the two treatments either (see also regression (3) in Table 2). That is, even when taking the passenger in the AV into account in the number of collision victims, we did not see a difference in the participants’ driving positions of the AV between the treatments *AV without* and *AV with passenger*.

**Table 2:** Coefficients and, in parentheses, standard errors of regressions on which Fig. 3b is based.

	Dependent variable: *distance to more passengers*		
	*(1)*	*(2)*	*(3)*
	*AV without passenger*	*AV with passenger*	*AV without vs. with passenger*
*Constant*	48.92*** (5.57)	52.88*** (5.27)	48.92*** (5.57)
*Ratio of passengers*	2.86 (2.72)	1.17 (2.57)	2.86 (2.72)
*AV with passenger* (= 1)			3.96 (7.66)
*(AV with passenger)* × *(ratio of passengers)*			− 1.69 (3.74)
Observations	305	290	595
Log likelihood	− 1321.64	− 1273.39	− 2595.52
AIC	2649.3	2552.8	5201.0

*p < 0.05 **p < 0.01 ***p < 0.001.

### Influence of the type of other road users

**Figure 4:**
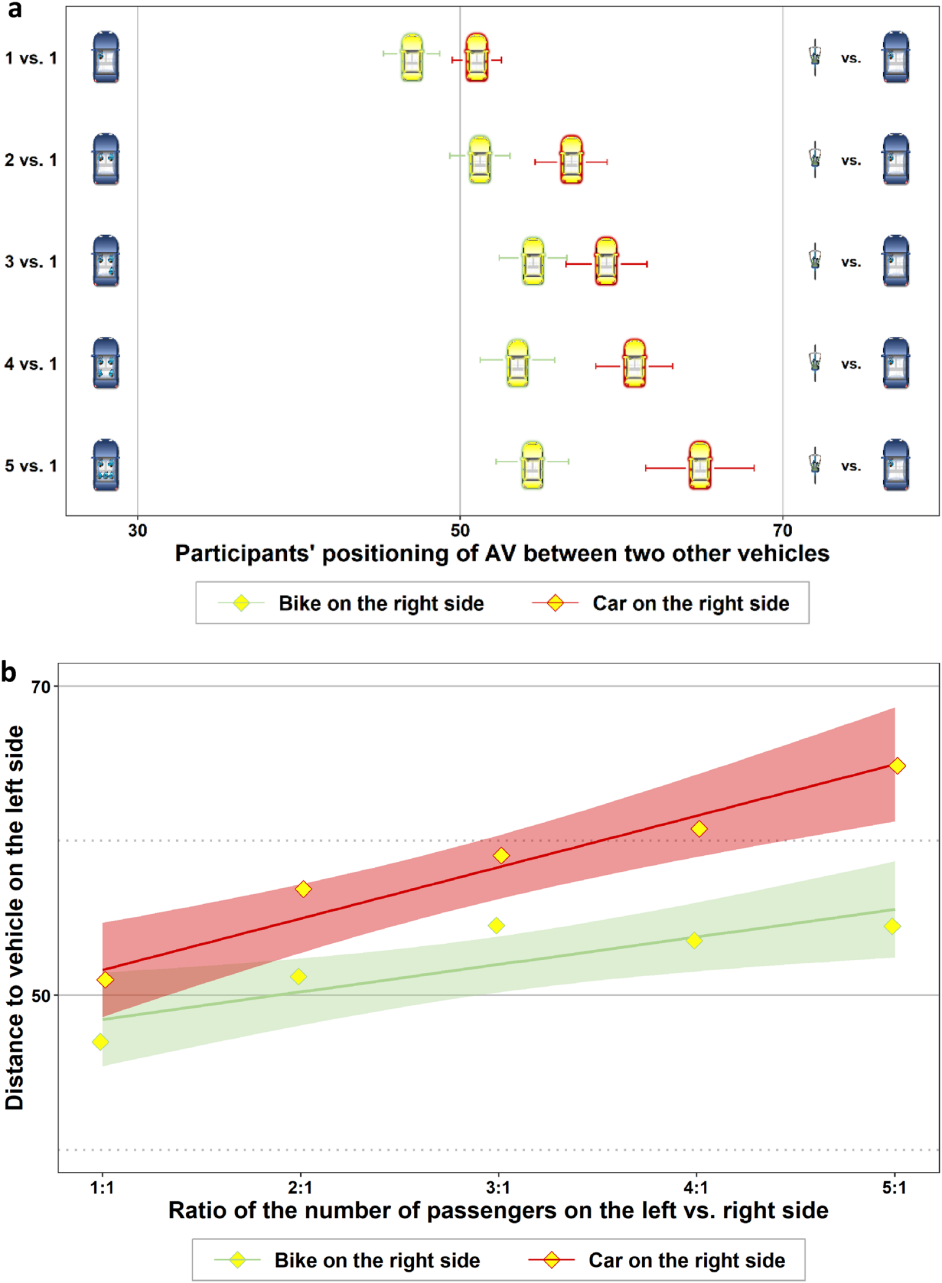
Risk allocation between different types of road users. (**a**) Means and standard errors of participants’ positioning of the AV with different numbers of passengers in the blue car on the left side and different road users on the right side. AVs with red frames depict the results when there was a car on the right side; those with green frames show the results when there was a cyclist on the right side. (**b**) The results of a linear regression in which the AV’s distance from the car on the left side was regressed on the increasing disproportion of passengers on the left and right sides. The disproportion of passengers was interacted with a dummy variable for the treatment (cyclist on the right yes/no). Red and green regions visualize 95% CIs.

Participants considered the number of potential collision victims in their lateral lane positioning of the AV. But do they also consider the type of other road users? Figure 4a shows participants’ average positioning of the AV for different vehicle types on the right side of the road. AVs with green frames show average positions when there was a cyclist, and red frames show average positions when there was a car with one passenger on the right side. If the AV traveled between a car with one passenger and one cyclist, the cyclist was granted marginally more safety distance at the expense of the car passenger (1 vs. 1: *t* = 1.72, *p* = 0.088). As the number of car passengers increased on the left side, the AV was positioned closer to the cyclist, just as if there was a car with one passenger on the right side of the road. In both these treatments, we see a significant positive trend between the AV’s safety distance to the car on the left side of the road and the number of passengers in that car (see Fig. 4b and regressions (1) and (2) in Table 3). This positive trend was similar in both treatments, but it was shifted slightly towards the left side of the road when there was a cyclist on the right side (see Fig. 4b and regression (3) in Table 3). This may indicate a small risk bonus attributed to the cyclist by the participants in our scenarios. Note that the cyclist’s risk bonus could not be attributed to higher vulnerability (for a consistent finding, see^18^) because a collision in our scenarios was always fatal for all involved parties; we verified that participants understood this. Normatively, a cyclist’s risk bonus could be justified with his or her lower imposition of traffic risks on other road users.

**Table 3:** Coefficients and, in parentheses, standard errors of regressions on which Fig. 4b is based.

	Dependent variable: *distance to more passengers*		
	*(1)*	*(2)*	*(3)*
	*Car on the right side*	*Bike on the right side*	*Car vs. bike on the right side*
*Constant*	48.32*** (1.98)	46.67*** (1.96)	48.32*** (1.98)
*Ratio of passengers*	3.32*** (0.75)	1.78** (0.64)	3.32*** (0.75)
*Bike on right side* (= 1)			− 1.65 (2.79)
*(Bike on right side)* × *(ratio of passengers)*			− 1.55 (0.98)
Observations	308	521	829
Log likelihood	− 1326.48	− 2325.7	− 3656.82
AIC	2659.0	4657.4	7323.6

*p < 0.05 **p < 0.01 ***p < 0.001.

### Follow-up study: smallest collision probability for AV

The finding that the participants were willing to accept small risks to themselves for the benefit of others when they were a passenger in the AV is surprising, especially in light of the identified “social dilemma of autonomous vehicles”^17^. To ensure that participants had indeed understood that the middle driving position between the two other vehicles minimized the probability of a collision for them as a passenger in the AV, we conducted a follow-up study. In this follow-up study, we re-invited a randomly selected subsample of the main study with the aim of recruiting 100 participants again. We implemented two treatments, both of which were based on the same traffic situation. In each case, the participants were passengers sitting in the (yellow) AV and there were five passengers in the (blue) car on the left and one passenger in the (blue) car on the right side of the road.

**Figure 5:**
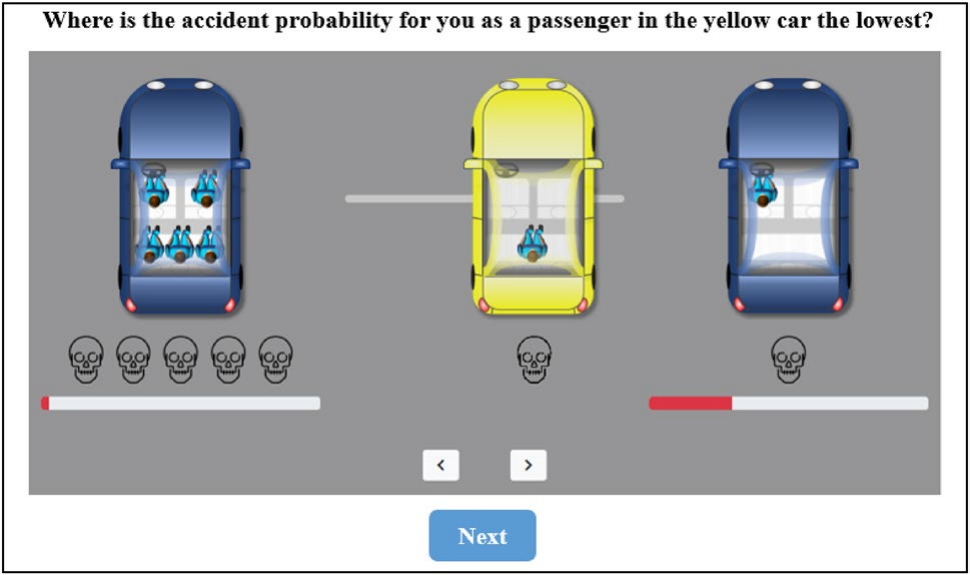
Graphical interface of slider task in follow-up study.

In one treatment, the participants could again adjust the driving position of the AV between the two other cars in 99 increments. As in the main study, the initial position of the yellow car was chosen at random for each participant. This time, however, we did not ask them where they thought the AV should drive, but where the probability of a collision was the lowest for them as a passenger in the (yellow) AV (see Fig. 5). The participants in this treatment (*n* = 51) positioned the AV exactly in the middle between the two other cars (*mean* = 49.5, *s.e.* = 1.75).

**Figure 6:**
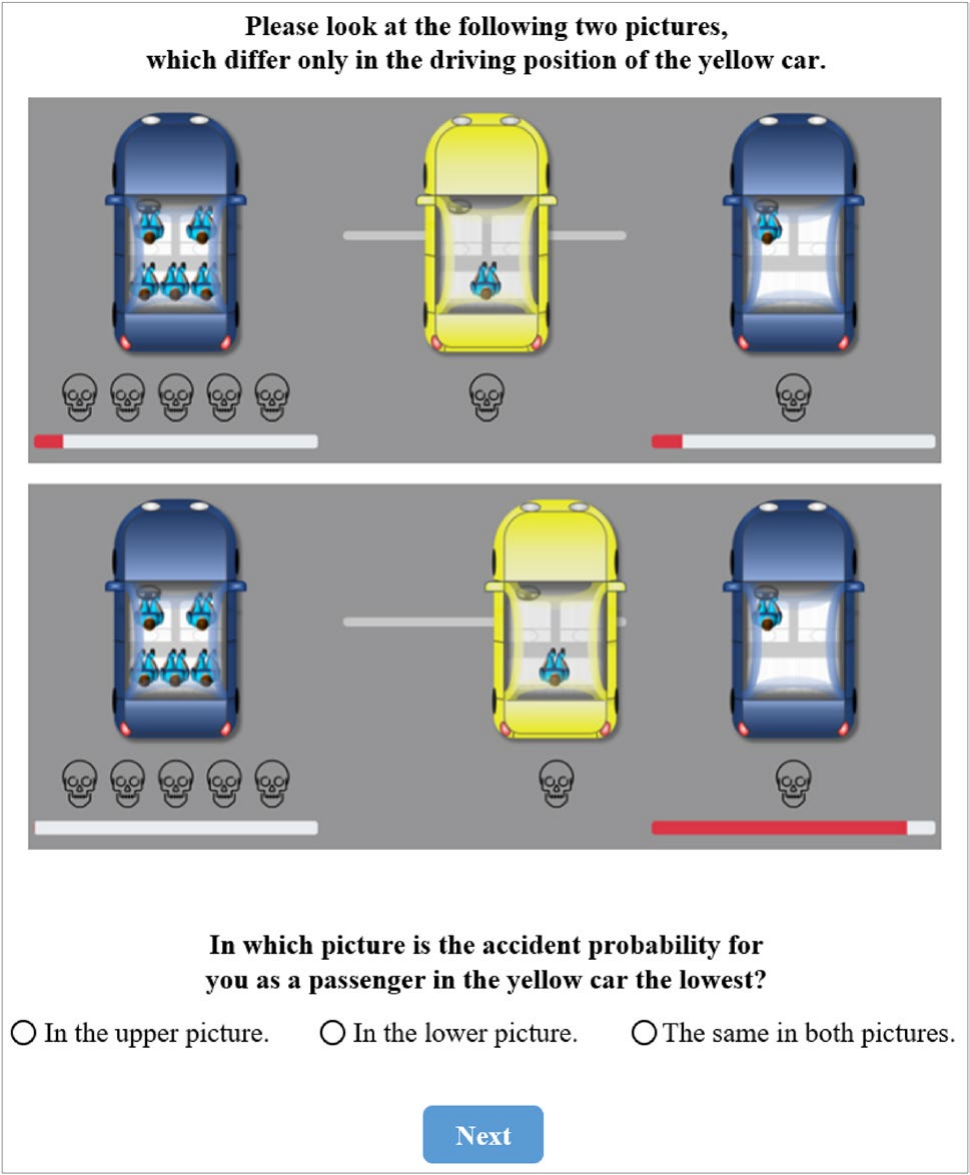
Single choice task in follow-up study.

In the other treatment of the follow-up study, participants could not position the AV themselves, but they saw two pictures with three answering options. In one picture, the AV was driving exactly between the other two cars, and in the other picture it was driving on the far right and thus closer to the blue car with one passenger (see Fig. 6). We asked the participants in this treatment (*n* = 57) in which of the two pictures the probability of a collision would be the lowest for them as a passenger in the AV. 50 participants (= 87.7%) chose the correct picture (i.e., the upper one), two of them (= 3.5%) chose the incorrect picture and five participants (= 8.8%) indicated that the probability would be the same in both pictures.

Finally, we asked all participants in both treatments on the last screen, without using any pictures, where the collision probability for the yellow car is the lowest in the described traffic situation. Participants could choose between (a) further to the left, (b) right in the middle between the two cars, (c) further to the right, and (d) the same everywhere. 99 participants (= 92%) chose the correct answer (b), two participants chose “further to the left,” four chose “further to the right”, and three chose “the same everywhere”. All in all, the results show that almost all participants of the follow-up study had understood where the lowest collision probability for the AV was in our traffic situation. Thus, it seems reasonable to assume that the participants in our main study consciously accepted risks and did not do so merely because of a faulty understanding of the traffic situation.

## Discussion and conclusion

Moral dilemmas in road traffic are not restricted to unavoidable collision scenarios that simply call for emergency braking^25–27^. Participation in everyday road traffic entails a distribution of risks between road users. This raises ethical questions, especially when this distribution is enforced by AVs. The patents from *Google* and others show that the car industry is thinking about the risk management of AVs and that their driving behavior could potentially be adjusted to account for the risks posed to other road users. The question is which variables should be included in this risk management and how AVs should then select the supposedly right maneuver from several possible ones. To reflect the moral intuitions of the German population, the driving algorithm would have to account for the number of potential collision victims and possibly the type of road users in determining AVs’ driving behavior. This is neglected if mere collision avoidance (explicitly or implicitly) determines the programming of driving algorithms.

Even if people are asked to imagine that they are passengers, they express their acceptance of a higher collision probability for themselves if this decreases the probability of a more severe collision for others. This is noteworthy with respect to the social dilemma of AVs identified in the context of unavoidable collisions^17^. There, people approved of utilitarian AVs and wanted others to buy them but would themselves prefer AVs that protect them at all costs. We find that people are more altruistic in the risky than in the deterministic domain. Understandably, one may be more willing to rescue a child from a burning building if one sees a chance of survival than if one is sure to die. This divergence in stated preferences provides another reason to shift the ethics of AVs from the extreme case of unavoidable collisions to the regular case of risk distributions in mundane traffic maneuvers. It is important, however, to replicate this finding in further research to corroborate that participants in our study actually identified with the passenger in the self-driving car. This could be done, for instance, by emulating personal involvement through an incentivized study in which participants lose some monetary endowment whenever “their” vehicle gets involved in a collision.

Because the complexities of risk pose a challenge to moral theory^17,28^, the question is how to deal with the unavoidable imposition of risk in road traffic. One view is that contract theories may be the most promising approach to the imposition of risk^12^. Under contract theories, an imposition of risk is justified if and only if the affected have agreed that it is acceptable. A driving algorithm whose training data is based on the value judgments of a representative population sample could be interpreted as an implementation of aggregate consensus. This would also correspond to a principle requiring that citizens are informed about the ethical implications of driving algorithms and that they are able to provide input into the respective decision-making process^29^. It might be argued that such value judgments have normative force because they are elicited before a concrete traffic situation materializes. In the heat of real situations, drivers’ local interests will naturally diverge from their normative principle of how safety distances *should* be chosen. In manual driving, local interests guide actual behavior and will thus always overrule reflected ideals. For the first time, AVs allow to close this motivational gap and to commit drivers on the road to their normative principles. This may be considered an ethical opportunity of AVs besides the more salient promise of higher traffic safety. Training the driving algorithm on the moral judgments of the citizens that are affected by AVs as obtained in a popular vote may be a possible approach, alternative to the implementation of a top-down normative principle. In any case, even if such a top-down principle is implemented, it seems reasonable to be aware of whether it resonates with people’s preferences or whether it diverges and is imposed on them for commercial or paternalistic reasons.

## Methods

The online survey was programmed with the software *oTree*^30^ and conducted in Germany in December 2021 with the help of the survey service provider *Cint* (https://www.cint.com/). A total of 1807 participants completed the survey and representativeness of this sample was ensured by the survey provider according to the variables age (between 18 and 69 years), gender and federal state. The study was executed according to the principles of the Declaration of Helsinki, and it was approved by the Institutional Review Board of the German Association for Experimental Economic Research (https://www.gfew.de). Prior to conducting the study, it was registered with full details, including the number of treatments, number of participants, and planned data analysis. The pre-registration can be accessed at the following link: https://aspredicted.org/i7xm8.pdf. Screenshots of the entire survey can be found in Appendix 2.

At the beginning of the survey, we obtained informed consent from all respondents to participate in the study. Subsequently, the traffic situation, their task and the graphical interface were described to the participants. Thereafter, two control questions about the described traffic situation had to be answered. Only participants who answered these two control questions correctly, qualified to take part in the rest of the survey. All other participants were screened out of the survey.

After correctly answering the control questions, the participants encountered the traffic situation in which they had to position the yellow AV between the two other vehicles as they deemed it to be correct. All participants were confronted with only one traffic situation and this situation was drawn at random out of 29 possible situations. In all situations, a (yellow) AV drove between two other vehicles. In 24 of the 29 situations, the vehicles on either side were cars, each with a varying number of passengers. In the remaining 5 situations, one cyclist was shown on the right and a car with varying numbers of passengers on the left. If the AV traveled between two cars, the participants were passengers in the AV themselves in some of these situations. Otherwise, the AV was always empty, i.e., without any passengers.

In total, we had 18 different combinations of the number of road users on the left and right sides of the road in our survey. There were five in the treatment with a cyclist on the right side (*Bike on the right side*), as the number of passengers in the car on the left side varied between one and five (i.e., we had the combinations 1 vs. 1, 2 vs. 1, 3 vs. 1, 4 vs. 1, 5 vs. 1). In the treatment in which the AV traveled without any passengers between two other cars (*AV without passenger*), we had eight different combinations (i.e., 1 vs. 1, 2 vs. 1, 3 vs. 1, 4 vs. 1, 5 vs. 1, 3 vs. 2, 4 vs. 2, 5 vs. 2). In the treatment in which the AV traveled with the participants as a passenger between two other cars (*AV with passenger*), we had five different combinations of the number of road users on the left and right sides of the road (i.e., 1 vs. 1, 2 vs. 1, 3 vs. 1, 4 vs. 1, 5 vs. 1). For each of the 18 different combinations of the number of road users on both sides of the road (eight in *AV without*, five in *AV with passenger*, five in *Bike on the right side*), we aimed for 100 participants. Because the 1807 participants were assigned at random to the traffic situations, we ended up with small variations in the number of observations per situation. In *AV without* and *AV with passenger*, we also controlled for the side of the road with more passengers (i.e., whether there were more passengers in the car on the left or right side of the road). This too was assigned at random, so that the number of observations in these situations was roughly split in half between the two possible variants. Table 4 shows all 29 traffic situations in our study and the respective number of observations.

**Table 4:**
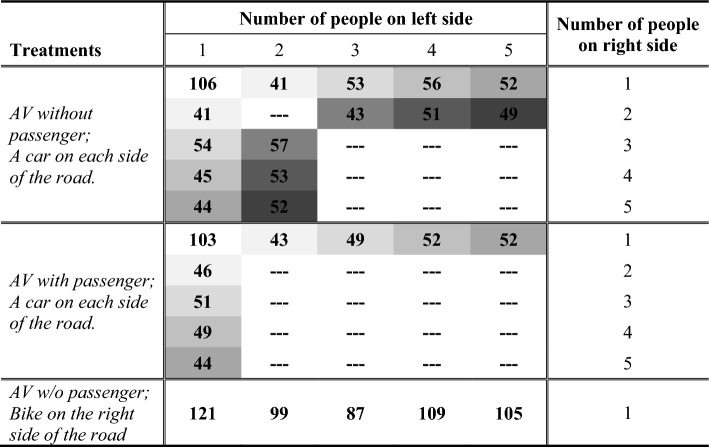
Number of observations in each traffic situation in our study.

Cells shaded alike in the same section of the table indicate paired traffic situations that differed only in terms of whether the majority of road users appeared on the left or right side of the road.

Following the positioning of the (yellow) AV, the participants answered a small questionnaire in which we asked about some demographic and personal characteristics. After that, the survey was completed. The median survey response time was about 4.5 min. Overall, we had 50.2% women, and the mean age of the participants was 44 years. 93% of the participants had a driver’s license and 78% reported driving a vehicle themselves on a regular basis (i.e., at least twice a week as a driver). With an average of 2.5, on a scale between 0 (“not at all”) and 6 (“very much”), the participants were not particularly looking forward to a future with self-driving cars. More details on the sample and a descriptive overview per treatment can be found in Table S1 in Appendix 1.

### Supplementary Information

Supplementary Information.

## Data Availability

The data will be made available upon request by the corresponding author of this publication.
