# Supplementary material for: The risk ethics of autonomous vehicles: an empirical approach
Source: Sci Rep. 2024 Jan 10;14:960. doi: 10.1038/s41598-024-51313-2 (PMC10781762; doi:10.1038/s41598-024-51313-2)
Supplement: Supplementary file 1 — Supplementary Information. [file 41598_2024_51313_MOESM1_ESM.pdf]

Appendix for

The risk ethics of autonomous vehicles: An empirical approach

Sebastian Krügel, Matthias Uhl

Appendix 1: Supporting information

Table S1 provides a descriptive overview of the participants in our study and shows that the randomized assignment to the treatments worked well. In each of the treatments, slightly more than half of the participants (approximately 55%) failed to answer the control questions correctly. We tolerated the high drop-out rate because it was important to us to check the participants' understanding of the task rather than just administering attention tests, which typically result in drop-out rates of 20% to 30%. The average age of participants in all treatments was approximately 44 years, and we had approximately equal proportions of men and women. Most participants (approximately 93%) possessed a driver's license, and almost half reported using a motorized vehicle as a driver more than five times per week. 12% to 13% of participants in each treatment reported having a job in a technological field, such as computer science or engineering. Because the academic literature in engineering often argues for accident avoidance as a guiding principle in road traffic (see, e.g., *10*) we wanted to examine whether we find a similar view among people in a technological job in our survey.

With 2.33 to 2.69 points, on a scale ranging from 0 ("not at all") to 6 ("very much"), participants indicated on average that they were not very excited about a future with self-driving cars. This question was the only one of the post-experimental questionnaire in which the treatments differed slightly but significantly. Participants in the treatment *Bike on the right side* were, on average, 0.3 points less excited about a future with self-driving cars than participants in the other two treatments. The idea of mixed road traffic with cyclists and self-driving cars in this treatment may have further dampened participants' excitement about a future with self-driving cars. Apart from that, our participants were very risk averse in all treatments. With a possible win of 100 euros with probability 0.5 in our hypothetical gamble, participants indicated on average that they would only be willing to pay about 12 to 13 euros to participate in such a gamble. Finally, we had asked two questions of the cognitive reflection test [*31*], since it is assumed in the literature that better performance in this test is associated with stronger utilitarian attitudes (see, e.g., [*32*]). The "bat and ball problem" was solved correctly by only about a quarter of our participants, and the "machine and widgets problem" by about half of them.

	Treatment		
	AV without passenger	*AV with passenger*	*Bike on the right side*
Total participants	1,837	1,128	1,140
Qualified participants (control questions)	797	489	521
Drop-out rate	56.6 %	56.6 %	54.3 %
Age (*in years*)	44.0	44.6	43.6
Male	48.4 %	51.5 %	49.9 %
Driver license	93.6 %	92.6 %	93.1 %
Driving a motor vehicle			
Never	11.2 %	12.1 %	12.5 %
Up to one time a week	9.8 %	9.4 %	11.3 %
Two to three times a week	14.4 %	15.5 %	13.4 %
Four to five times a week	17.8 %	17.0 %	14.6 %
More than five times a week	46.8 %	46.0 %	48.2 %
In favor of a speed limit on highways			
Yes	56.0 %	54.2 %	54.3 %
No	37.1 %	37.0 %	39.0 %
Don't know	6.9 %	8.8 %	6.7 %
Job in technology sector	13.7 %	11.9 %	12.3 %
Happiness about future with AVs	2.60	2.69	2.33^{**}
Risk attitude (*in euros*)	13.65	12.21	12.27
Correct "bat and ball" problem	25.5 %	24.9 %	25.5 %
Correct "machine and widgets" problem	49.6 %	53.2 %	51.4 %

$^{*}p<0.05$ $^{**}p<0.01$ $^{***}p<0.001$

Table S1: Descriptive overview of the participants in our study per treatment

Table S2 shows regression results based on all data for each treatment (*AV without passenger*, *AV with passenger*, *Bike on the right side*) when we added some demographic variables of our participants to each regression as well as the decision time (*in seconds*) for the task of positioning the AV and the initial (random) position of the AV. The dependent variable was the distance to the vehicle with more passengers in each case. As in the main text, the regressions showed that the greater the disproportion of passengers in the two vehicles on the left and right sides of the road, the greater the safety distance to the vehicle with more passengers. In addition, in treatment *AV without passenger*, we saw a significant restraint in deviating from the lane's middle when there were more passengers on the right, as opposed to the left side of the road. Possibly, this peculiarity stems from an internalization of the emergency lane, which in Germany, as well as in some other European countries, must always be formed between the far left lane and the other lanes. Apart from this restraint, only the "bat and ball problem" of the cognitive reflection test [*31*] was significantly associated with AV positioning. Participants who solved this problem correctly incorporated the number of passengers more strongly into their decision and were thus somewhat more utilitarian, which is generally consistent with conjectures in the literature (see, e.g., [*32*]). Other demographic

variables, the participants' risk attitude, their happiness about a future with AVs, their decision time in positioning the AV, and the randomized initial position of the AV did not affect the final positioning between the two other vehicles.

	Dependent variable: *Distance to more passengers*					
	AV without passenger		*AV with passenger*		*Bike on the right side*	
Constant	47.81^{***}	(3.22)	46.46^{***}	(4.27)	41.38^{***}	(4.87)
Ratio of passengers	2.32^{***}	(0.60)	2.03^{***}	(0.57)	1.77^{**}	(0.64)
More passengers on right side (= 1)	-3.61^{**}	(1.38)	−3.71	(1.90)		
Decision time	−0.02	(0.04)	0.00	(0.01)	0.00	(0.03)
Initial position of AV	0.03	(0.02)	0.02	(0.03)	0.03	(0.03)
Male (= 1)	−1.26	(1.42)	2.23	(1.72)	−3.47	(2.01)
Age (in years)	0.05	(0.05)	0.00	(0.06)	0.11	(0.07)
Job in technology sector (= 1)	2.55	(1.78)	3.66	(2.78)	0.15	(3.09)
No driver license (= 1)	−0.35	(3.73)	5.20	(3.34)	2.59	(3.61)
Happiness about future with AVs	−0.16	(0.40)	0.32	(0.51)	0.14	(0.61)
Risk attitude	−0.01	(0.04)	−0.04	(0.06)	0.02	(0.07)
"Bat and ball probl." (Correct = 1)	3.14^{*}	(1.39)	−1.25	(1.62)	0.07	(1.97)
Observations	795		489		521	
Log Likelihood	−3,445.16		−2,104.55		−2,322.30	
AIC	6,916.3		4,235.1		4,668.6	

$^{*}p<0.05$ $^{**}p<0.01$ $^{***}p<0.001$

Table S2: Coefficients and, in parentheses, standard errors of regressions for each treatment with the AVs' safety distance to more passengers as dependent variable.
In treatment *AV without passenger*, two observations were excluded in which the corresponding participants indicated that they were neither male nor female.

Appendix 2: Screens used in the survey

Below you will find all the screens of the survey. The texts on the screens are translated from German into English.

Information on the purpose of this research and informed consent

Purpose:

This survey is conducted for the purpose of academic research. As part of the study, we are interested in your moral judgments about the driving behavior of self-driving cars.

Procedure:

Participation in this study involves completing a short questionnaire. We anticipate that it will take approximately seven to ten minutes to complete the questionnaire. To participate, you must be at least 18 years old.

Confidentiality:

All of your responses will be anonymous.

Are you voluntarily taking part in this survey?

- o Yes
- o No

Next

Fig. S1. Screen 1: Informed consent.

Your task

This is about a possible future traffic situation in which self-driving cars participate in regular road traffic. It is expected that self-driving cars will increase overall road safety. However, even with self-driving cars, the following rule applies: The smaller the distance to other road users, the higher the risk of a collision due to unforeseeable events.

As the car increases the distance to one road user, it often decreases the distance to another road user in the process. The risks of a collision are very small, but not zero. Should the self-driving car account for the number of potential crash victims when weighing these risks?

Your task is to position the self-driving car in a possible traffic situation as **you** deem appropriate. The self-driving yellow car **without passengers** is driving between two other vehicles. **Where do you think the self-driving car should drive: right in the middle between the two vehicles, further to the left, or further to the right?**

The closer the self-driving car is to the left or right vehicle, the higher the probability of a collision with that vehicle. **If a collision occurs, everyone involved in the collision is dead!** The yellow self-driving car cannot collide with both vehicles at the same time.

In the following, a traffic situation is shown as an example. This situation is for you to play around with and is not included in the survey.

Example situation: Please try it out! Drag the car around or use the arrow keys below.

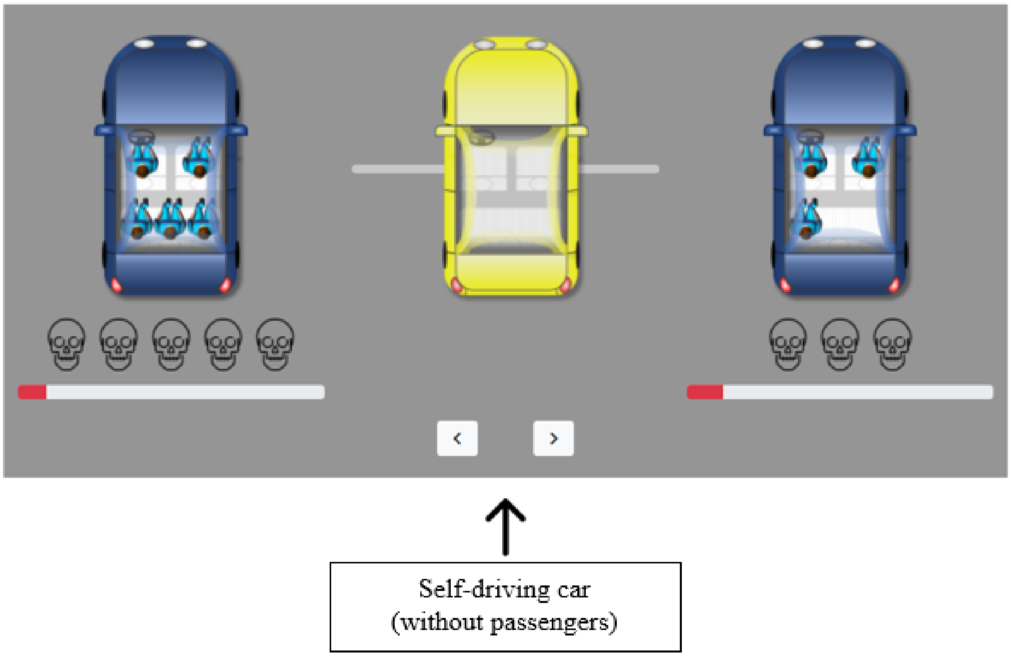

On the next page, we will ask you two comprehension questions about your task. Only those who answer these questions correctly can continue with the survey.

Next

Fig. S2. Screen 2a: Description of the traffic situation, the task and graphical interface (treatments *AV without passenger* and Bike *on the right side*).

Your task

This is about a possible future traffic situation in which self-driving cars participate in regular road traffic. It is expected that self-driving cars will increase overall road safety. However, even with self-driving cars, the following rule applies: The smaller the distance to other road users, the higher the risk of a collision due to unforeseeable events.

As the car increases the distance to one road user, it often decreases the distance to another road user in the process. The risks of a collision are very small, but not zero. Should the self-driving car account for the number of potential crash victims when weighing these risks?

Your task is to position the self-driving car in a possible traffic situation as **you** deem appropriate. The self-driving yellow car **with you as a passenger** is driving between two other vehicles. **Where do you think the self-driving car should drive: right in the middle between the two vehicles, further to the left, or further to the right?**

The closer the self-driving car is to the left or right vehicle, the higher the probability of a collision with that vehicle. **If a collision occurs, everyone involved in the collision is dead!** The yellow self-driving car <u>cannot</u> collide with both vehicles at the same time.

In the following, a traffic situation is shown as an example. This situation is for you to play around with and is not included in the survey.

Example situation: Please try it out! Drag the car around or use the arrow keys below.

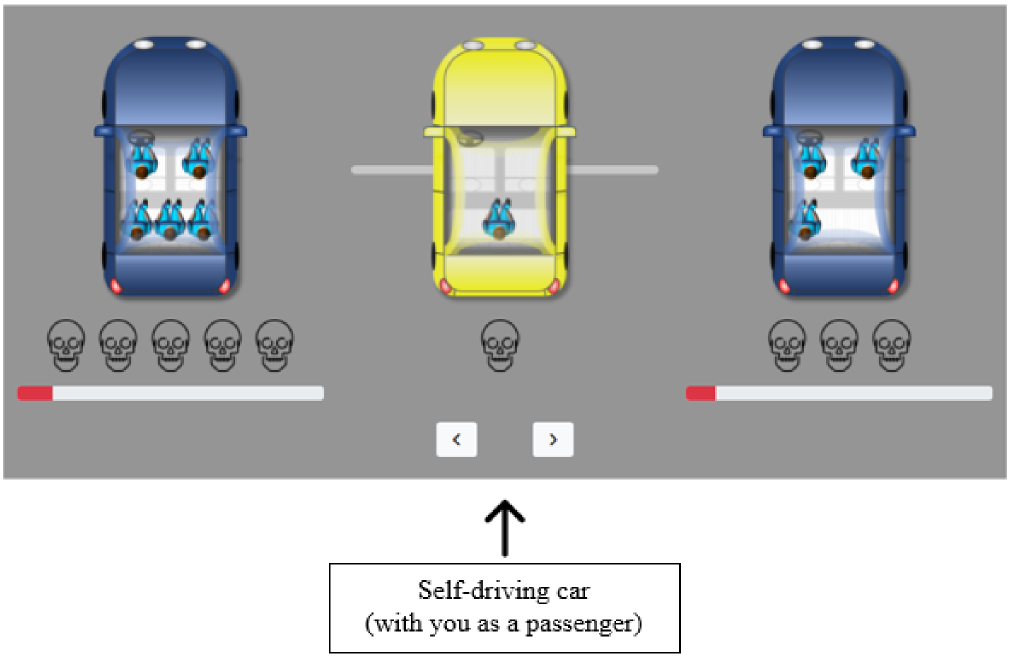

On the next page, we will ask you two comprehension questions about your task. Only those who answer these questions correctly can continue with the survey.

Next

Fig. S3. Screen 2b: Description of the traffic situation, the task and graphical interface (treatments *AV with passenger*).

Comprehension questions

How high is the risk of a collision in the traffic situation we have described?

- o Very low, but not zero.
- o A collision with one of the vehicles is certain.

If a collision occurs, we assume that everyone involved in the collision is dead.

- o True
- o False

Next

Fig. S4. Screen 3: Both control questions (all treatments).

You answered all questions correctly!

Now please indicate for the traffic situation where you think the yellow self-driving car should drive by dragging it to the left or right.

Next

Fig. S5. Screen 4: Survey continues if both control questions were answered correctly.

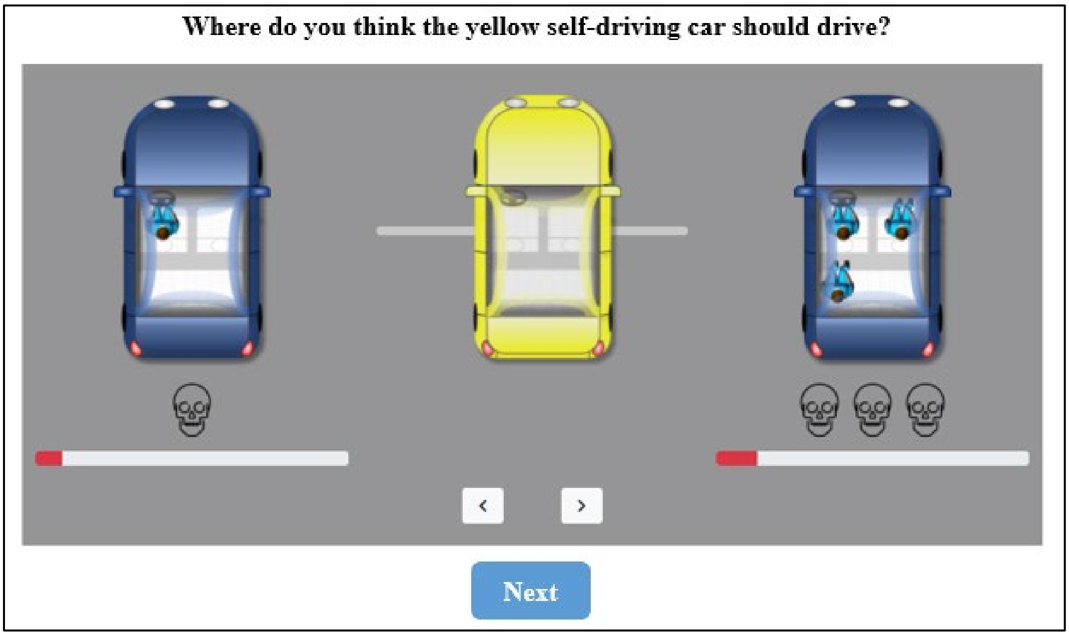

Fig. S6. Screen 5a: Decision screen (treatments *AV without passenger*).

Participants were able to drag the (yellow) AV back and forth between both other vehicles in 99 increments. The initial position of the AV was chosen at random for each participant. The red bar below each vehicle visualized the collision probability as a function of the distance of the AV. The closer the AV to a vehicle, the greater [smaller] the probability of a collision with that [the opposite] vehicle. The red bar below each vehicle increased [decreased] accordingly. We implemented an exponential relationship between the distance of the AV and the collision probability with a vehicle according to the following function, where the middle driving position of the AV between the two other vehicles minimized the overall accident probability:

$P(collision) = \left(\frac{1}{\sqrt[8]{x}} - 0.562\right) \times 10^{-2}$ with $x \in \{1, 2, 3, \dots, 99\}$ representing the distance of the AV to the respective vehicle.

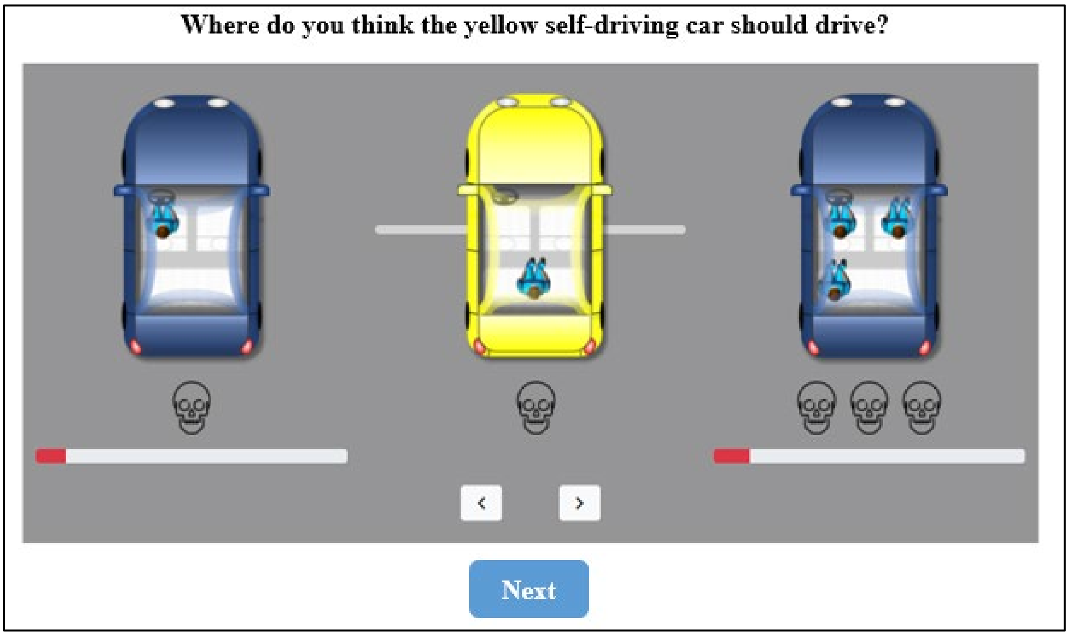

Fig. S7. Screen 5b: Decision screen (treatments *AV with passenger*).

(*Also see the notes to Fig. S6.*)

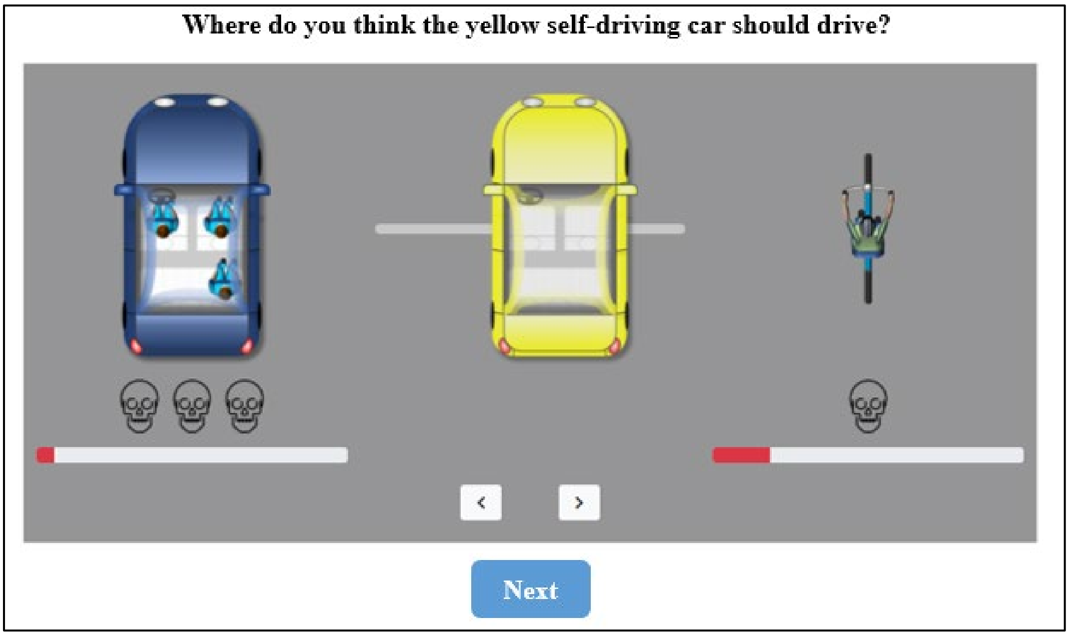

Fig. S8. Screen 5c: Decision screen (treatments *Bike on the right side*).

(*Also see the notes to Fig. S6.*)

To conclude the survey, we ask you to answer a few more questions.

In which year were you born?

____

What is your gender?

- Female.
- Male.
- Other.

In which state do you live?

[Dropdown menu: A list of all states in Germany.]

To which occupational field do you most closely associate your occupational activity?

[Dropdown menu: Agriculture, forestry, animal husbandry; Technology, computer science, engineering; Commercial profession, trade, tourism, administration; Health, social services, teaching; Military; Other field; Not employed.]

Next

Fig. S9. Screen 6: Demographic and personal characteristics – 1.

To conclude the survey, we ask you to answer a few more questions.

Do you have a driver's license?

- Yes.
- No.

How regularly do you usually operate a motor vehicle as a driver?

[Dropdown menu: More than five times a week; Four to five times a week; Two to three times a week; Up to one time a week; Never]

Would you vote for a general speed limit on highways in Germany?

- Yes.
- No.
- Don't know.

Next

Fig. S10. Screen 7: Demographic and personal characteristics – 2.

To conclude the survey, we ask you to answer a few more questions.

Which party would you vote for if there was a federal election next Sunday?

[Dropdown menu: SPD; CDU/CSU; Die Grünen; FDP; AFD; Die Linke; Other]

Are you looking forward to a future with self-driving cars?

[Dropdown menu: 0 (not at all); 1; 2; 3 (in between); 4; 5; 6 (very much)]

Next

Fig. S11. Screen 8: Demographic and personal characteristics – 3.

To conclude the survey, we ask you to answer a few more questions.

Now imagine that there is a lottery in which an ordinary coin is tossed. In case of "heads" you get 0 euros, in case of "tails" you get 100 euros. What would you be willing to pay to participate in the lottery?

[A slider between 0 and 100 €]

Next

Fig. S12. Screen 9: Risk attitude based on hypothetical task.

To conclude the survey, we ask you to answer a few more questions.

A bat and a ball cost 1.10 € in total. The bat costs 1.00 € more than the ball. How much does the ball cost?

____________ Cents

If it takes 5 machines 5 minutes to make 5 widgets, how long would it take 100 machines to make 100 widgets?

____________ Minutes

Next

Fig. S13. Screen 10: Two questions of the cognitive reflection test.

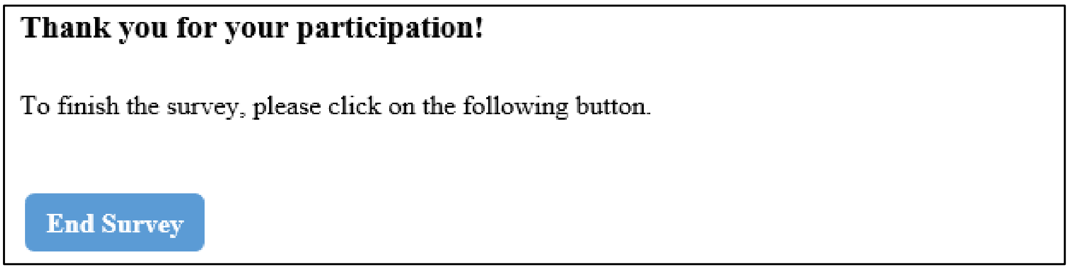

Fig. S14. Screen 11: End of survey.

References

31. S. Frederick, Cognitive Reflection and Decision Making. *Journal of Economic Perspectives* **19**(4), 25-42 (2005).

32. J. Greene, *Moral tribes: Emotion, reason, and the gap between us and them*. Penguin (2014).
